# Treatment Patterns and Survival of Patients With Advanced Non-Small Cell Lung Cancer Guided by Comprehensive Genomic Profiling: Real-World Single-Institute Study in China

**DOI:** 10.3389/fonc.2021.630717

**Published:** 2021-03-10

**Authors:** Weize Lv, Hua Cheng, Di Shao, Yajun Wei, Weiping Zhu, Kui Wu, Wenxi Jiang, Liyang Hu, Zhou Sha, Beilong Zhong, Xiaofeng Pei

**Affiliations:** ^1^ Department of Interventional Medicine, Fifth Affiliated Hospital of Sun Yat-sen University, Zhuhai, China; ^2^ Guangdong Provincial Key Laboratory of Biomedical Imaging, Fifth Affiliated Hospital of Sun Yat-sen University, Zhuhai, China; ^3^ Department of Cardiothoracic Surgery, Fifth Affiliated Hospital Sun Yat-sen University, Zhuhai, China; ^4^ BGI Genomics, BGI-Shenzhen, Shenzhen, China; ^5^ Department of Nephrology, Fifth Affiliated Hospital of Sun Yat-sen University, Zhuhai, China; ^6^ BGI-Shenzhen, Shenzhen, China; ^7^ Department of Thoracic Oncology, The Fifth Affiliated Hospital of Sun Yat-sen University, Zhuhai, China

**Keywords:** non-small cell lung cancer, targeted therapy, real-world evidence, comprehensive genomic profiling, survival

## Abstract

Although the National Comprehensive Cancer Network and the Chinese Society of Clinical Oncology guidelines recommend comprehensive genomic profiling of lung adenocarcinoma, it has not been widely applied in Chinese hospitals. This observational study aimed to determine real-world evidence of whether comprehensive genomic profiling can benefit the survival of patients with lung cancer. We investigated the frequency of genomic alterations, treatment strategies, and clinical outcomes in 233 patients with advanced non-small cell lung carcinoma who were routinely screened using a 508-gene panel. The most prevalent drivers were mutations of EGFR (51%), KRAS (9%), PIK3CA (7%), ALK (7%), MET (6%), and BRAF (5%). Mutations in tumor suppressor genes included TP53, KEAP1, RB1, PTEN, and APC. Median overall survival (OS) was significantly shorter among patients harboring KRAS (mutant, n = 17; WT, n = 154) and TP53 (mutant, n = 103; WT n =68) mutations (11.3 *vs.* 24.0 months; P = 0.16 and 18.7 *vs.* 28.7 months; P = 0.018, respectively). The OS was longer among patients with tumors harboring EGFR (P = 0.069) and ALK (P = 0.51) mutations. Most patients (65.4%) with the driver gene-positive (EGFR, ALK, and ROS1) tumors were received TKI treatment, whereas those with driver gene wild tumors (53.1%) chose platinum-based therapy. Univariate and multivariate analyses associated a shorter OS among patients with tumors harboring concomitant TP53 and EGFR mutations. These findings provide additional evidence from real-world on the potential importance of targeted therapies as a treatment option in NSCLC patients harboring clinically actionable mutation.

## Introduction

According to the guidelines of the National Comprehensive Cancer Network, molecular tests of epidermal growth factor receptor (EGFR), anaplastic lymphoma kinase (ALK), c-ros oncogene 1 (ROS1), B-Raf Proto-Oncogene (BRAF), mesenchymal-epithelial transition (MET), and rearranged during transfection (RET) are recommended for all non-small cell lung cancers (NSCLC) if specimens are sufficient for molecular analysis (1). The Chinese Society of Clinical Oncology recommends tests for EGFR mutations as well as ALK and ROS1 fusion (category 1) in non-squamous NSCLC and next-generation sequencing to detect all clinically meaningful mutations (category 2) (2). Resistance to targeted agents also requires changes in treatment strategies through repeated biopsies and molecular tests; thus, molecular analysis of advanced NSCLC has become routine (3). The French National Cancer Institute (INCa) launched a nationwide program for the routine molecular profiling of patients with advanced non-squamous NSCLC at 28 certified molecular genetics centers (4). The results suggested that routine nationwide molecular profiling provided a clinical benefit to patients with advanced NSCLC (5). In 2014, the Lung Cancer Mutation Consortium published their first study (LCMC1) reporting on mutation data of 1,007 late-stage NSCLC patients evaluated at 14 institutions in United States, this study demonstrated multiplexed testing aided physicians in selecting therapies and patients for targeted trials (6). However, a similar nationwide project does not exist in China, where clinicopathological and genomic data are retained in hospitals and third-party testing institutions, respectively. These data should be combined to generate real-world evidence (RWE) that supports comprehensive genomic profiling and promotes the application of routine molecular profiling in more hospitals.

The emergence of targeted therapy has redefined the treatment strategies of driver gene-positive advanced lung cancer (7–10). These targeted agents have been approved based on the results of randomized controlled trials (RCTs). Drugs are undoubtedly effective in ideal populations defined by RCTs, but the effectiveness of these drugs in actual clinical practice requires further verification. Real-world studies (RWSs) summarize the diagnosis and therapeutic data of clinical patients and provide generalizable evidence for precise medicine interventions, thus overcoming the inadequacy of RCT conclusions that are limited to specific patients (11). In addition, RCTs of patients with lung cancers harboring particularly rare mutations, such as EGFR exon 20 insertions and RET-NTRK fusions, are difficult to implement. RWSs are designed to meet clinical needs and can include most oncology patients, accelerating patient recruitment (12). This single-center, prospective, observational study aimed to determine the feasibility of routine molecular profiling and the clinical benefits for patients with advanced NSCLC.

## Materials and Methods

### Participants and Data Collection

The Institutional Review Board of Fifth Affiliated Hospital of Sun Yat-Sen University approved this study. Written informed consent was obtained from each of 233 patients with NSCLC or their legal representatives. We then collected epidemiological and clinicopathologic data (age, sex, smoking status, date of disease diagnosis, TNM stage, and dates of disease progression). Overall survival (OS) was calculated as the interval between diagnosis and death or final follow-up. The cutoff for outcomes was May 2020.

### Comprehensive Genomic Profiling

Genomic analysis was performed using Oseq, which is a clinical test designed to detect mutations, copy number alterations, and select gene fusions among 508 cancer-associated genes (13). Genomic DNA (50–200 ng) or cell-free DNA (10–50 ng) extracted from formalin-fixed paraffin-embedded (FFPE) or plasma samples were processed to generate bar-coded libraries according to manufacturer’s instructions (Integrated DNA Technologies, Coralville, IA, USA). Then exons were captured using IDT custom-designed probes (Integrated DNA Technologies, Coralville, IA, USA). A control library was constructed using DNA from white blood cells to filter germline mutations. DNA sequencing was performed on MGISEQ-2000 sequencing system (MGI, Shenzhen, China). Genomic alterations including base substitutions, short insertions and deletions, copy number alterations, and gene fusions were detected using a customized analysis pipeline which is modified from The Genome Analysis Toolkit (14). Data interpretation was focused on genomic alterations associated with clinically available targeted treatment options according to the standards and guidelines of the NCCN, the Association for Molecular Pathology (AMP), the American Society of Clinical Oncology (ASCO) and the College of American Pathologists (CAP) (15). The tumor mutation burden (TMB) was calculated as the number of non-synonymous mutations in non-driver genes per sample divided by the genomic coverage for that sample (1.7 Mb). The median TMB served as the cutoff to define TMB-high and TMB-low samples. Microsatellite instability (MSI) status was determined using MSIsensor and MANTIS (16, 17).

### Statistical Analysis

Categorical variables were compared using χ² tests or Fisher’s exact tests. The median age was compared between groups using Wilcoxon rank sum tests. The date of the initial diagnosis of lung cancer until the date of death or final follow-up was defined as OS, which was estimated from Kaplan-Meier curves. Differences in OS were analyzed using log-rank tests. Independent effects of concurrent mutations were assessed using multivariable Cox proportional hazards models adjusted for age, sex, and smoking history. Data were statistically analyzed and figures were prepared using R 3.6.3 (R Foundation for Statistical Computing, Vienna, Austria) with a significance level of 0.05.

## Results

### Characteristics of the Patients

We obtained 321 test reports of routine comprehensive genomic profiling between January 2016 and October 2019. After excluding 88 of other histologic types, 233 patients with NSCLC were included in the study. **Table 1** summarizes their clinical characteristics. The median age was 61 (IQR, 51–67) years, and 43.1% were female. Formalin-fixed paraffin-embedded tissues and plasma samples were obtained from 177 (76.3%) and 55 (23.7%) patients, respectively, for genomic profiling. The most prevalent histological subtype was lung adenocarcinoma (198, 85.0%). Most patients had stage IV tumors (200, 85.8%). The TMB significantly differed between females and males, and between smokers and non-smokers (P = 0.001), but not according to age or tumor stage (**Table S2**).

**Table 1:** Patient characteristics (N = 233).

Characteristic	No. (%)
**Gender**	
Female	101 (43.3%)
Male	132 (56.7%)
**Age at diagnosis, y**	
Median	61.0
Q1, Q3	51.0, 67.0
**Histology**	
Adenocarcinoma	198 (85.0%)
Squamous cell carcinoma	35 (15.0%)
**Stage**	
III	33 (14.2%)
IV	200 (85.8%)
**Sample type**	
Plasma	55 (23.6%)
Tissue	178 (76.4%)
**TMB status**	
Low	116 (49.8%)
High	117 (50.2%)
**MSI**	
MSS	146 (80.7%)
MSI-L	35 (19.3%)
Unknown	52
**Smoking history**	
History of smoking	74 (33.0%)
No history of smoking	150 (67.0%)
Unknown	9
**Status of Survive**	
Alive	107 (50.5%)
Deceased	105 (49.5%)
Unknown	21

### Distribution of Mutated Genes

**Figure 1:**
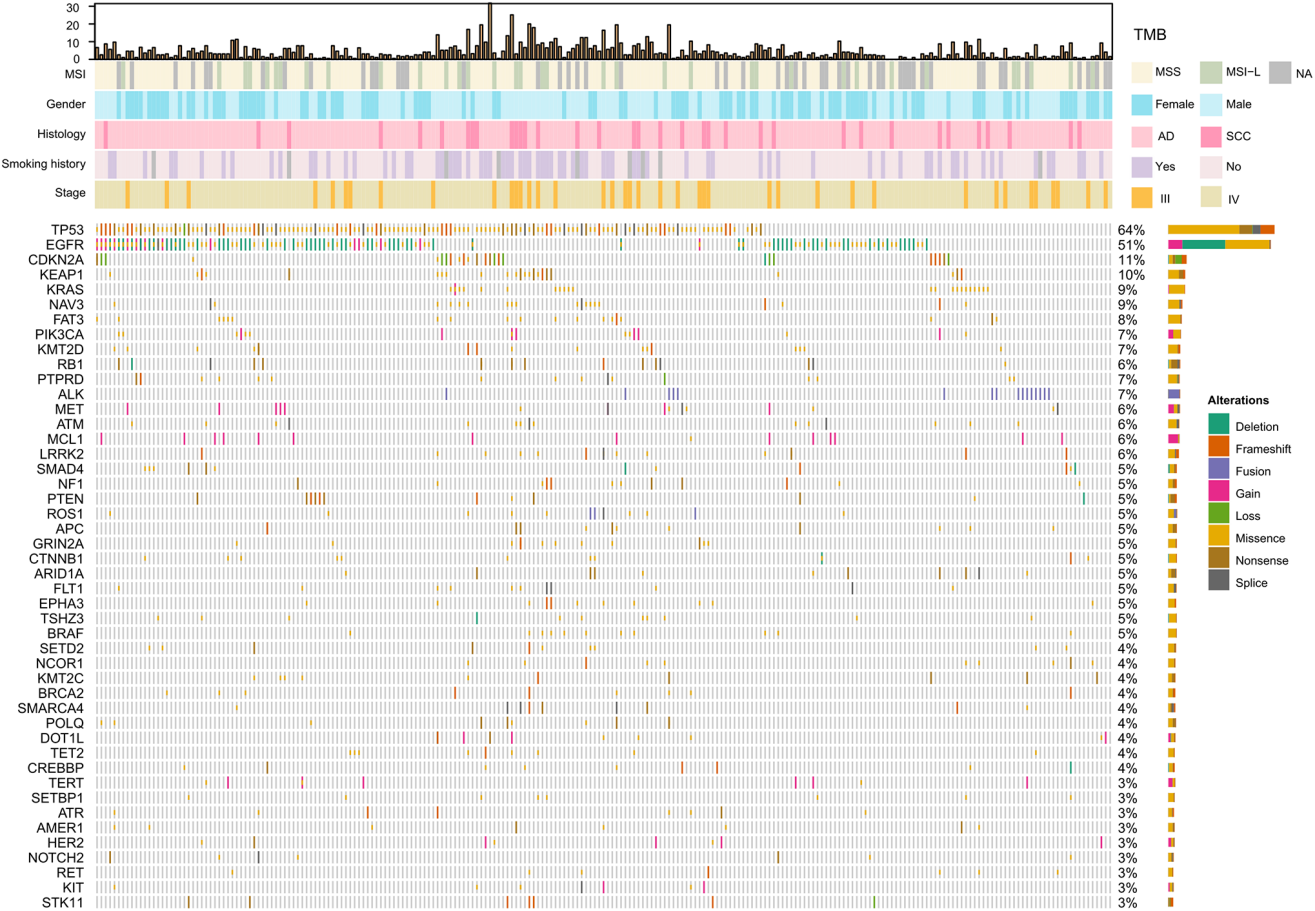
Significantly mutated genes and clinicopathological features of 233 patients with NSCLC. TMB, tumor mutation burden; MSS, microsatellite stability; MSI-L, Microsatellite instability—low; NA, not available; AD, adenocarcinoma; SCC, squamous cell carcinoma.

The spectrum of significantly mutated genes was similar to that in our previous study (18). The most common driver mutations were in EGFR (51%) followed by KRAS (9%), PIK3CA (7%), ALK (7%), MET (6%), and BRAF (5%) (**Figure 1**). Mutations were present in tumor suppressor genes including TP53, KEAP1, RB1, PTEN, and APC. ALK fusions, HER2 insertions, MEK mutations, KIF5B-RET, and CD74-ROSI were mutually exclusive. **Figure S1** compares mutation in significantly mutated genes in adenocarcinoma between this cohort and The Cancer Genome Atlas (TCGA) lung adenocarcinoma cohort (n = 515). Notable differences from TCGA data included EGFR (51.1 *vs* 13.0%, P < 0.001), KRAS (9.4 *vs* 30.0%; P < 0.001), CDKN2A (10.7 *vs* 4.0%, P < 0.001), KEAP1 (9.9 *vs* 19%, P < 0.001), NAV3 (8.6 *vs* 20%, P < 0.001), FAT3 (8.1 *vs* 21%, P < 0.001), PTPRD (6.9 *vs* 17%, P < 0.001), and NF1 (5.1 *vs* 12%, P = 0.005). High TMB was more common in young women and smokers (P = 0.001) (**Table S2**). Genomic alterations in TP53, EGFR, BRAF, ROSI, and RET were associated with significantly higher TMB than wild types cases (**Figure S2**).

We also analyzed associations between driver alterations and clinical characteristics. EGFR mutations were more common in women(65.3 *vs* 34.7%; P <.001), and non-smokers (61.3 *vs* 35.1%; P <. 001) (**Table 2**). The mutation rate of KRAS was significantly higher in men than in women (12.9 *vs* 5.0%, P = 0.04) and in heavy smokers than in non-smokers (17.6 *vs* 6.0%, P = 0.006) (**Table 2**). Patients with ALK rearrangements were more often younger (mutant *vs* WT: 53.0 *vs* 61.0 years; P = 0.003) (**Table 2**).

**Table 2:** EGFR, KRAS, ALK, ROS1, and MET mutation status stratified by clinical characteristics.

	EGFR			KRAS			ALK			ROS1			MET		
	mut (N = 119)	wild (N = 114)	p value^†^	mut (N = 22)	wild (N = 211)	p value^†^	mut (N = 16)	wild (N = 217)	p value^†^	mut (N = 12)	wild (N = 221)	p value^†^	mut (N = 15)	wild (N = 218)	p value^†^
**Gender**			<0.001			0.040			0.312			0.055			0.418
Female	66 (65.3%)	35 (34.7%)		5 (5.0%)	96 (95.0%)		5 (5.0%)	96 (95.0%)		2 (2.0%)	99 (98.0%)		5 (5.0%)	96 (95.0%)	
Male	53 (40.2%)	79 (59.8%)		17 (12.9%)	115 (87.1%)		11 (8.3%)	121 (91.7%)		10 (7.6%)	122 (92.4%)		10 (7.6%)	122 (92.4%)	
**Age at diagnosis**			0.318			0.194			0.003			0.779			0.562
Median	58.5	62.0		64.5	59.0		53.0	61.0		58.000	61.000		62.500	60.000	
Q1, Q3	49.7, 66.0	54.0, 69.0		58.0, 69.7	51.0, 66.0		45.2, 56.2	52.0, 69.0		54.5, 62.5	51.0, 67.0		48.750, 72.000	51.000, 67.000	
**Histology**			<0.001			0.413			0.081			0.321			0.850
AD	111 (56.1%)	87 (43.9%)		20 (10.1%)	178 (89.9%)		16 (8.1%)	182 (91.9%)		9 (4.5%)	189 (95.5%)		13 (6.6%)	185 (93.4%)	
SCC	8 (22.9%)	27 (77.1%)		2 (5.7%)	33 (94.3%)		0 (0.0%)	35 (100.0%)		3 (8.6%)	32 (91.4%)		2 (5.7%)	33 (94.3%)	
**Stage**			0.059			0.291			0.122			0.460			0.086
IV	104 (53.9%)	89 (46.1%)		20 (10.4%)	173 (89.6%)		11 (5.7%)	182 (94.3%)		9 (4.7%)	184 (95.3%)		10 (5.2%)	183 (94.8%)	
III	15 (37.5%)	25 (62.5%)		2 (5.0%)	38 (95.0%)		5 (12.5%)	35 (87.5%)		3 (7.5%)	37 (92.5%)		5 (12.5%)	35 (87.5%)	
**MSI**			0.812			0.165			0.946			0.043			0.106
MSS	70 (47.9%)	76 (52.1%)		15 (10.3%)	131 (89.7%)		12 (8.2%)	134 (91.8%)		7 (4.8%)	139 (95.2%)		9 (6.2%)	137 (93.8%)	
MSI-L	16 (45.7%)	19 (54.3%)		1 (2.9%)	34 (97.1%)		3 (8.6%)	32 (91.4%)		5 (14.3%)	30 (85.7%)		5 (14.3%)	30 (85.7%)	
**Smoking history**			<0.001			0.006			0.340			0.027			0.245
Yes	26 (35.1%)	48 (64.9%)		13 (17.6%)	61 (82.4%)		3 (4.1%)	71 (95.9%)		7 (9.5%)	67 (90.5%)		7 (9.5%)	67 (90.5%)	
No	92 (61.3%)	58 (38.7%)		9 (6.0%)	141 (94.0%)		11 (7.3%)	139 (92.7%)		4 (2.7%)	146 (97.3%)		8 (5.3%)	142 (94.7%)	
**TMB status**			<0.001			0.077			0.116			0.018			0.803
low	75 (64.7%)	41 (35.3%)		7 (6.0%)	109 (94.0%)		11 (9.5%)	105 (90.5%)		2 (1.7%)	114 (98.3%)		7 (6.0%)	109 (94.0%)	
high	44 (37.6%)	73 (62.4%)		15 (12.8)	102 (87.2%)		5 (4.3%)	112 (95.7%)		10 (8.5%)	107 (91.5%)		8 (6.8%)	109 (93.2%)	

^†^Pearson’s Chi-squared test or Linear Model ANOVA was used for distribution of cohort characteristics between male and female groups.

### Prognostic Implications of Clinical and Genomic Features

**Figure 2:**
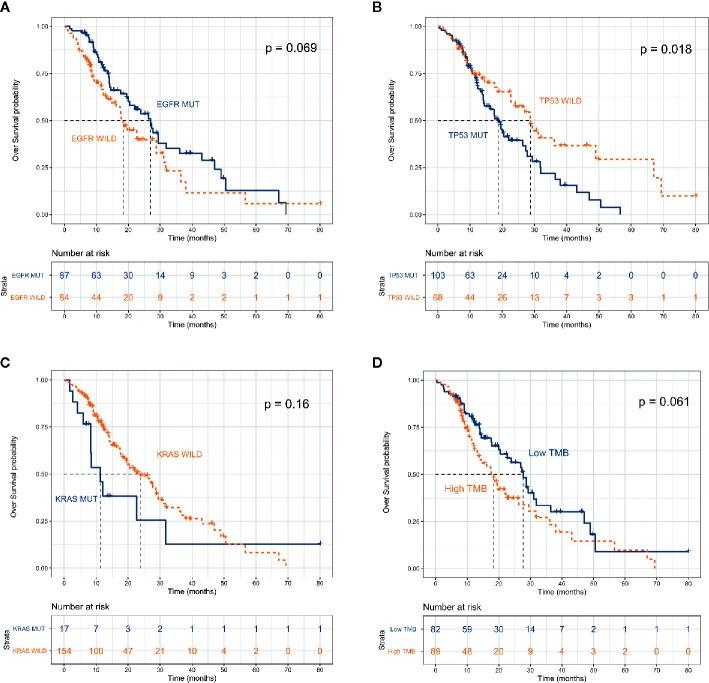
Overall survival in patients with advanced non–small cell lung cancer (n = 171) stratified by mutation status of EFGR **(A)**, TP53 **(B)** and KRAS **(C)** and tumor mutation burden **(D)**.

The median follow-up of 171 patients with appropriate data and advanced disease was 14.4 (IQR 9.4–22.8) months. The mOS from initial diagnosis was 22.7 (95% CI, 17.7–28.7) months (**Figure S3A**). OS did not significantly differ between smokers and non-smokers (mOS: 19.6 *vs* 26.4 months; P = 0.54) (**Figure S3C**). Although the OS was longer for patients with adenocarcinoma (n = 146) than SCC (n = 25), the difference did not reach statistical significance (mOS: 24.0 *vs* 18.4 months; P = 0.57) (**Figure S3D**). We compared survival between patients with or without driver mutations. Patients harboring KRAS (mutant, n = 17; WT, n = 154) and TP53 (mutant, n = 103; WT, n = 68) mutations had a shorter OS (mOS: 11.3 *vs* 24.0 months; P = 0.16, and 18.7 *vs* 28.7 months, P = 0.018, respectively); whereas among patients harboring a driver mutation for which a targeted therapy was available, OS was longer for those whose tumors harbored EGFR (P = 0.069) and ALK (P = 0.51) mutations (**Figures 2A–C** and **Figure S4A**). Analysis of the potential prognostic implications of immune-related biomarkers associated a low TMB with longer OS (mOS: 18.4 *vs* 27.7 months; P = 0.061); OS did not significantly differ between low MSI (n = 27) and microsatellite stability (MSS) (n = 112) groups (P = 0.61) (**Figure 2D** and **Figure S4D**).

### Treatment Patterns and Clinical Outcomes

**Figure 3:**
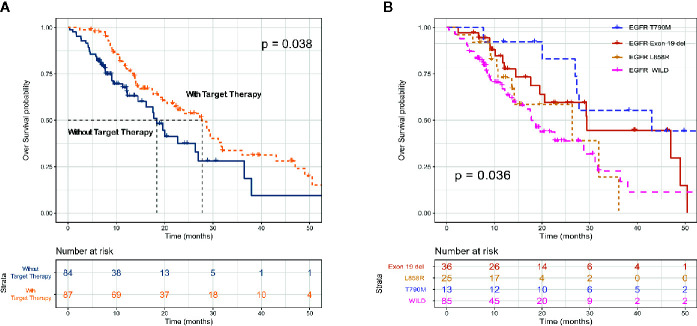
Kaplan–Meier survival curves for overall survival according to receipt of targeted therapy (EGFR TKI and ALK TKI) **(A)** and EGFR mutation subtype **(B)**.

All advanced patients (n = 171) received first-line therapy. Most patients with driver gene-negative tumors (53.1%) were treated with platinum-based regimens, whereas most patients with mutated tumors (EGFR, ALK, and ROS1) (65.4%) were treated with TKIs. **Table 3** shows the details of the treatment regimens. Most (66 [64.3%] of 87) patients with mutated EGFR were treated with the EGFR TKIs, gefitinib (38 [24.1%]), erlotinib (n = 3), icotinib (n = 4), and afatinib (n = 11). Similarly, most patients (8 [61.5%] of 13) with ALK fusions were treated with the ALK TKIs crizotinib, alectinib, and lorlatinib. OS was longer for patients treated with TKIs (EGFR, ALK, and ROS1) than with platinum-based chemotherapy (mOS: 27.7 *vs* 18.4 months; p = 0.038; **Figure 3A**). Survival outcomes in response to EGFR TKIs notably differed among exon 19 deletions, L858R mutations, and acquired resistance T790M in EGFR. The mOS was the longest and second longest among patients with an acquired T790M mutation and exon 19 del, respectively, and the shortest among patients with L858R point mutations (**Figure 3B**).

**Table 3:** First-line therapy pattern of patients with actionable genomic alteration.

	EGFR (N = 87)	KRAS (N = 17)	ALK (N = 13)	ROS1 (N = 7)	MET (N = 5)	Full-wild^**^ (N = 42)	Total (N = 171)
Platinum-based Chemo	21 (24.1%)	8 (47.1%)	4 (30.8%)	1 (14.3%)	1 (20.0%)	25 (59.5%)	60 (35.1%)
Gefitinib	38 (43.7%)	3 (17.6%)	0 (0.0%)	0 (0.0%)	0 (0.0%)	5 (11.9%)	46 (26.9%)
Erlotinib	3 (3.4%)	0 (0.0%)	0 (0.0%)	0 (0.0%)	0 (0.0%)	0 (0.0%)	3 (1.8%)
Icotinib	4 (4.6%)	1 (5.9%)	0 (0.0%)	0 (0.0%)	0 (0.0%)	0 (0.0%)	5 (2.9%)
Afatinib	11 (12.6%)	0 (0.0%)	0 (0.0%)	1 (14.3%)	0 (0.0%)	2 (4.8%)	14 (8.2%)
Crizotinib	1 (1.1%)	0 (0.0%)	5 (38.5%)	4 (57.1%)	3 (60.0%)	1 (2.4%)	14 (8.2%)
Alectinib	0 (0.0%)	1 (5.9%)	2 (15.4%)	0 (0.0%)	0 (0.0%)	2 (4.8%)	5 (2.9%)
Lorlatinib	0 (0.0%)	0 (0.0%)	1 (7.7%)	0 (0.0%)	0 (0.0%)	0 (0.0%)	1 (0.6%)
Other^*^	9 (10.3%)	4 (23.5%)	1 (7.7%)	1 (14.3%)	1 (20.0%)	7 (16.7%)	23 (13.5%)

*Including another type of chemotherapy, anti-VEGF agents, immunotherapy, or clinical trial therapies.

**Full-wild = patients without genomic alteration in EGFR, KRAS, MET, ALK, or ROS1.

### EGFR TKI Resistance and Concurrent Mutations

**Figure 4:**
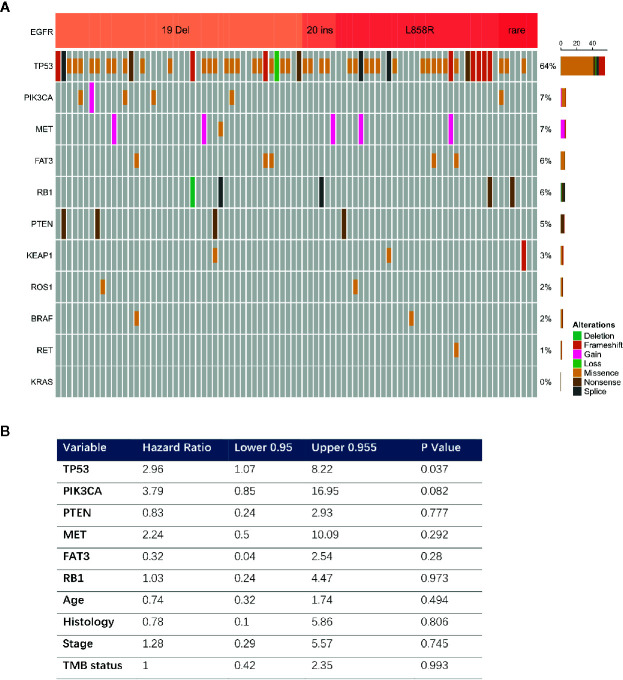
Concurrent genomic alteration with EGFR mutation and treatment efficacy of EGFR-TKIs. **(A)** Prevalence of concurrent genes in EGFR mutant patients (n = 86). **(B)** Cox Multivariable analysis of overall survival among patients with concurrent genomic alteration treaded with EGFR TKI.

Comprehensive genomic profiling identified 86 EGFR mutated tumors among 171 patients with advanced cancers and complete follow-up information. Most of these were exon 19 deletions (n = 44, 51.1%) or L858R mutations (n = 29, 33.7%). The most prevalent concurrent mutations were TP53 in 64% and PIK3CA in 7.0% of tumors with EGFR mutations (**Figure 4A**). We further analyzed the clinical outcomes and possible resistance mechanisms among patients with concurrent mutations treated with EGFR TKIs. Univariate analysis associated a shorter OS with a concurrent mutation in TP53 (HR, 2.96; 95% CI, 1.07–8.22; P = 0.037). A PIK3CA mutation was also associated with a difference in OS, but the difference was statistically significant (HR, 3.79; 95% CI, 0.85–16.95; P = 0.08). Multivariable Cox proportional hazard models (adjusted for TP53, PIK3CA, age, sex, and stage) associated TP53 with a shorter treatment duration (HR, 0.37; 95% CI, 0.98–7.81; P = 0.05, **Figure 4B** **)**.

## Discussion

The introduction of targeted therapy has changed the therapeutic landscape for lung cancers over the past decade (19). New targeted agents are continuously being approved by the FDA, and increasing numbers of patients are benefiting from precision medicines. The present study found that 62% of patients with NSCLC harbored actionable genetic alterations, meaning that tumor progression and metastasis could be managed with targeted drugs that also improved their quality of life. Precision medicine is the future of lung cancer management, and the primary prerequisite is a genomic map of tumors. Although the traditional molecular pathology detection methods (ARMS, IHC, and FISH) have been routinely applied, their limitations have become more obvious in the face of increasing numbers of targets. The emergence of high-throughput sequencing has provided a new tool for clinical molecular pathology detection. The simultaneous detection of all mutations related to therapy and prognosis is cost-effective and not only provides clinicians with a panoramic genetic map of cancer but also avoids the invasive collection of multiple clinical samples from patients. Based on the current evidence, ESMO recommends routine use of NGS on tumor samples in advanced NSCLC to accelerate cancer research and drug development through clinical trials, provide access to innovation to patients and to collect data (20). The turnaround time from specimen acquisition to clinical reports of assay findings at our molecular pathology laboratory is 6 days. Genomic reports provide clinicians with all information needed about possible sensitivity and resistance to targeted drugs. In addition to drug targets, our study proved that comprehensive genomic profiling also enabled the discovery of some mutations that can predict prognosis, such as TP53 mutation was associated with shorter survival, which is consistent with the findings of other studies (21, 22). The present findings provide RWE that comprehensive genomic profiling will benefit the survival of patients with lung cancer.

The EGFR 19 exon deletion (19del) and the 21 exon L858R mutation (21L858R) are classic sensitive mutations, but tumors with such mutations respond differently to TKIs. The ARCHER 1050 study of the second-generation EGFR TKI, dacomitinib, and the FLAURA study of the third-generation EGFR TKI, osimertinib, found that progression-free survival was longer for patients with 19del than with 21L858R (23, 24). Our subgroup analysis generated similar findings, indicating that better treatment options are needed for patients with EGFR 21L858R. The NEJ026 and CTONG 1509 study suggested that the anti-angiogenesis + EGFR TKI could confer a survival benefit upon patients with L858R-positive tumors (25, 26). Various TKIs have different effects on rare EGFR mutations such as E18, G719X, E21, L861Q, and E20 S768I. The objective response rate was better for the second-generation EGFR TKI agent afatinib than the first-generation agents gefitinib/erlotinib (27). The efficacy of both first- and second-generation EGFR-TKI is significantly lower than that of conventional chemotherapy for patients with E20 insertion mutations (28). Besides, it is founded that TP53 mutation and other oncogenic concurrent mutations were associated with worse OS in patients with EGFR mutant NSCLC (29–31). Therefore, patients with EGFR mutations require accurate molecular diagnosis before starting individualized therapy.

Acquired drug resistance in targeted therapy has always been an obstacle to improving the long-term survival of patients. Repeated biopsies after drug resistance are highly significant. Acquired drug resistance mutations can be monitored in real time, so physicians can make timely adjustments to treatment regimens. Replacing EGFR TKIs is the most direct strategy when the EGFR on-target resistance mechanism occurs. Osimertinib can be administered when a T790M mutation is acquired, and combined first- and third-generation TKIs can be administered when the osimertinib resistance mutation C797S is detected (32). Patients with EGFR TKI resistance caused by the activation of other driver genes can be treated with combined targeted therapies; for example, savolitinib+EGFR TKI can overcome EGFR TKI resistance caused by MET amplification (33). Patients in our cohort with acquired T790M mutations treated with osimertinib had the longest OS.

This study has some limitations. Although the rate of actionable genomic alterations was higher in our cohort than in the Caucasian population, the proportion of our patients treated with targeted drugs was lower than that in other studies. This is partly due to the fact that there were not many targeted drugs included in medical insurance when the patient received treatment. The CFDA has so far approved only the EGFR and ALK TKIs, gefitinib, erlotinib, afatinib, osimertinib, crizotinib, ceritinib, and alectinib, and these are covered by medical insurance. This issue will be resolved when more targeted drugs are approved such as capmatinib, selpercatinib, and lorlatinib to treat ROS1, MET exon 14 skipping, and RET fusion (34). Secondly, physicians will be able to recommend patients with more mutation-positive clinical and pathological features for comprehensive genomic testing. This selection bias will increase the proportions of specific mutations. Our comprehensive genomic testing also detected biomarkers that are related to the effects of immunotherapy such as TMB, MSI, and immune positively and negatively related gene mutations. However, because of the exorbitant cost of immunotherapy, only eight patients received PD-1/PD-L1 inhibitors. Therefore, we did not analyze correlations between these biomarkers and the effects of immune checkpoint inhibitors.

## Conclusions

In summary, our findings presented a clear genomic landscape of the mutation frequencies of oncogenic drivers from 233 patients with NSCLC and their associations with sex, smoking status, and TMB. These findings provide additional RWE about the potential importance of targeted treatment options for patients with NSCLC harboring clinically actionable mutations.

## Data Availability Statement

The datasets presented in this study can be found in online repositories. The names of the repository/repositories and accession number(s) can be found below: The data of this project has been submitted to CNGBdb (https://db.cngb.org/). The accession number of this project in CNGBdb is CNP0001436.

## Ethics Statement

The studies involving human participants were reviewed and approved by the Institutional Review Board of Fifth Affiliated Hospital of Sun Yat-Sen University. The patients/participants provided their written informed consent to participate in this study.

## Author Contributions

WL, BZ, and DS participated in study conception and design. XP, HC, YW, WZ, and LH enrolled and managed patients. XP, HC, DS, WJ and ZS carried out collection and assembly of data. DS and WL were involved in data analysis and interpretation. DS prepared the manuscript and manuscript figures. WL and KW edited, critically read, and revised the manuscript. All authors contributed to the article and approved the submitted version.

## Conflict of Interest

DS and DJ is a current employee of BGI Genomics.

The remaining authors declare that the research was conducted in the absence of any commercial or financial relationships that could be construed as a potential conflict of interest.
