# Supplementary material for: Treatment Patterns and Survival of Patients With Advanced Non-Small Cell Lung Cancer Guided by Comprehensive Genomic Profiling: Real-World Single-Institute Study in China
Source: Front Oncol. 2021 Mar 10;11:630717. doi: 10.3389/fonc.2021.630717 (PMC7988081; doi:10.3389/fonc.2021.630717)
Supplement: Supplementary file 1 [file DataSheet_1.docx]

# Supplementary Data

Table S1. Clinical Characteristics of Patients Stratified by Gender

Table S2. Clinical features of patients stratified by TMB status

Figure S1. comparison of mutations in significantly mutated genes in adenocarcinoma between this cohort (n=233) and The Cancer Genome Atlas (TCGA) lung adenocarcinoma cohort (n=515)

Figure S2. Median TMB between presence or absence of specified genetic mutation Supplement

Figure S3. Impact of clinical features on overall survival. Kaplan-Meier survival analysis in 171 patients with advanced non–small-cell lung cancer (A) stratified by age (B), smoking history (C), and histology (D).

Figure S4. Overall survival of patients stratified by presence of mutation in specified gene

Table S1. Clinical Characteristics of Patients Stratified by Gender

| **Characteristic** | Female (N=100) | Male (N=132) | Total (N=232) | p value ^†^ |
| --- | --- | --- | --- | --- |
| **Age at diagnosis** |  |  |  | 0.255 |
| Median | 58.000 | 61.000 | 61.000 |  |
| Q1, Q3 | 49.0, 66.0 | 54.0, 68.7 | 51.0, 67.0 |  |
| **Histology** |  |  |  |  |
| Adenocarcinoma | 88 (88.0%) | 109 (82.6%) | 197 (84.9%) |  |
| Squamous cell carcinoma | 12 (12.0%) | 23 (17.4%) | 35 (15.1%) |  |
| **Stage** |  |  |  | 0.088 |
| IV | 88 (88.0%) | 105 (79.5%) | 193 (83.2%) |  |
| III | 12 (12.0%) | 27 (20.5%) | 39 (16.8%) |  |
| **Sample type** |  |  |  | 0.045 |
| Plasma | 30 (30.3%) | 25 (18.9%) | 55 (23.8%) |  |
| Tissue | 69 (69.7%) | 107 (81.1%) | 176 (76.2%) |  |
| **TMB status** |  |  |  | 0.001 |
| Low | 62 (62.0%) | 54 (40.9%) | 116 (50.0%) |  |
| High | 38 (38.0%) | 78 (59.1%) | 116 (50.0%) |  |
| **MSI** |  |  |  | 0.645 |
| MSS | 56 (78.9%) | 89 (81.7%) | 145 (80.6%) |  |
| MSI-L | 15 (21.1%) | 20 (18.3%) | 35 (19.4%) |  |
| Unknown | 29 | 23 | 52 |  |
| **Smoking history** |  |  |  | < 0.001 |
| History of smoking | 4 (4.1%) | 70 (56.0%) | 74 (33.2%) |  |
| No history of smoking | 94 (95.9%) | 55 (44.0%) | 149 (66.8%) |  |
| Unknown | 2 | 7 | 9 |  |
| **Status of Survive** |  |  |  | 0.633 |
| Alive | 44 (48.4%) | 62 (51.7%) | 106 (50.2%) |  |
| Deceased | 47 (51.6%) | 58 (48.3%) | 105 (49.8%) |  |
| N-Miss | 9 | 12 | 21 |  |

^†^ Pearson's Chi-squared test or Linear Model ANOVA was used for distribution of cohort characteristics between male and female groups.

Table S2. Clinical features of patients stratified by TMB status

|  | TMB low (N=116) | TMB high (N=117) | Total (N=233) | p value^†^ |
| --- | --- | --- | --- | --- |
| **Gender** |  |  |  | 0.001 |
| Female | 62 (53.4%) | 39 (32.8%) | 101 (43.1%) |  |
| Male | 54 (46.6%) | 78 (67.2%) | 132 (56.9%) |  |
| **Age at diagnosis** |  |  |  | 0.009 |
| Median | 57.0 | 62.5 | 61.0 |  |
| Q1, Q3 | 48.0, 65.0 | 55.0, 69.0 | 51.0, 67.0 |  |
| **Histology** |  |  |  |  |
| Adenocarcinoma | 106 (91.4%) | 92 (78.6%) | 198 (85.0%) |  |
| Squamous cell carcinoma | 10 (8.6%) | 25 (21.4%) | 35 (15.0%) |  |
| **Stage** |  |  |  | 0.311 |
| IV | 99 (85.3%) | 94 (80.3%) | 193 (82.8%) |  |
| III | 17 (14.7%) | 23 (19.7%) | 40 (17.2%) |  |
| **Sample type** |  |  |  | 0.144 |
| Plasma | 32 (27.6%) | 23 (19.7%) | 55 (23.6%) |  |
| Tissue | 84 (72.4%) | 94 (80.3%) | 178 (76.4%) |  |
| **MSI** |  |  |  | 0.231 |
| MSS | 67 (77.0%) | 79 (84.0%) | 146 (80.7%) |  |
| MSI-L | 20 (23.0%) | 15 (16.0%) | 35 (19.3%) |  |
| Unknown | 29 | 23 | 52 |  |
| **Smoking history** |  |  |  | < 0.001 |
| History of smoking | 25 (22.1%) | 49 (44.1%) | 74 (33.0%) |  |
| No history of smoking | 88 (77.9%) | 62 (55.9%) | 150 (67.0%) |  |
| Unknown | 3 | 6 | 9 |  |

^†^ Pearson's Chi-squared test or Linear Model ANOVA was used for distribution of cohort characteristics between male and female groups.





Figure S1. comparison of mutations in significantly mutated genes in adenocarcinoma between this cohort (n=233) and The Cancer Genome Atlas (TCGA) lung adenocarcinoma cohort (n=515)


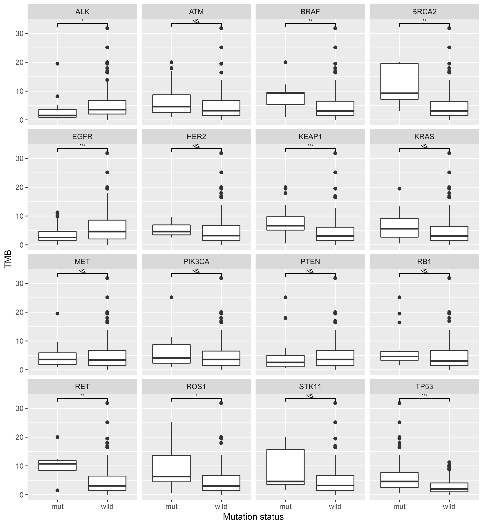


Figure S2. Median TMB between presence or absence of specified genetic mutation Supplement


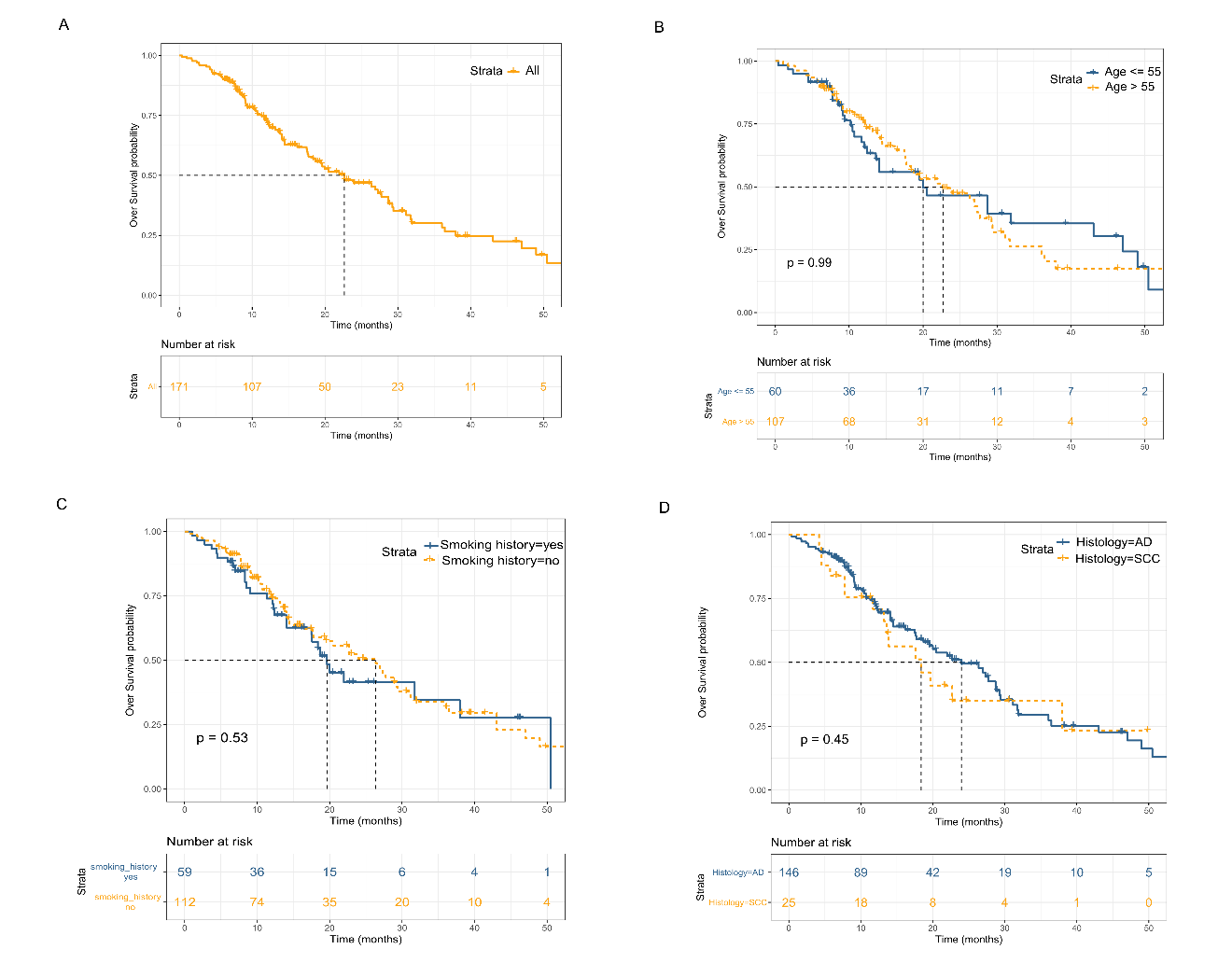


Figure S3. Impact of clinical features on overall survival. Kaplan-Meier survival analysis in 171 patients with advanced non–small-cell lung cancer (A) stratified by age (B), smoking history (C), and histology (D).


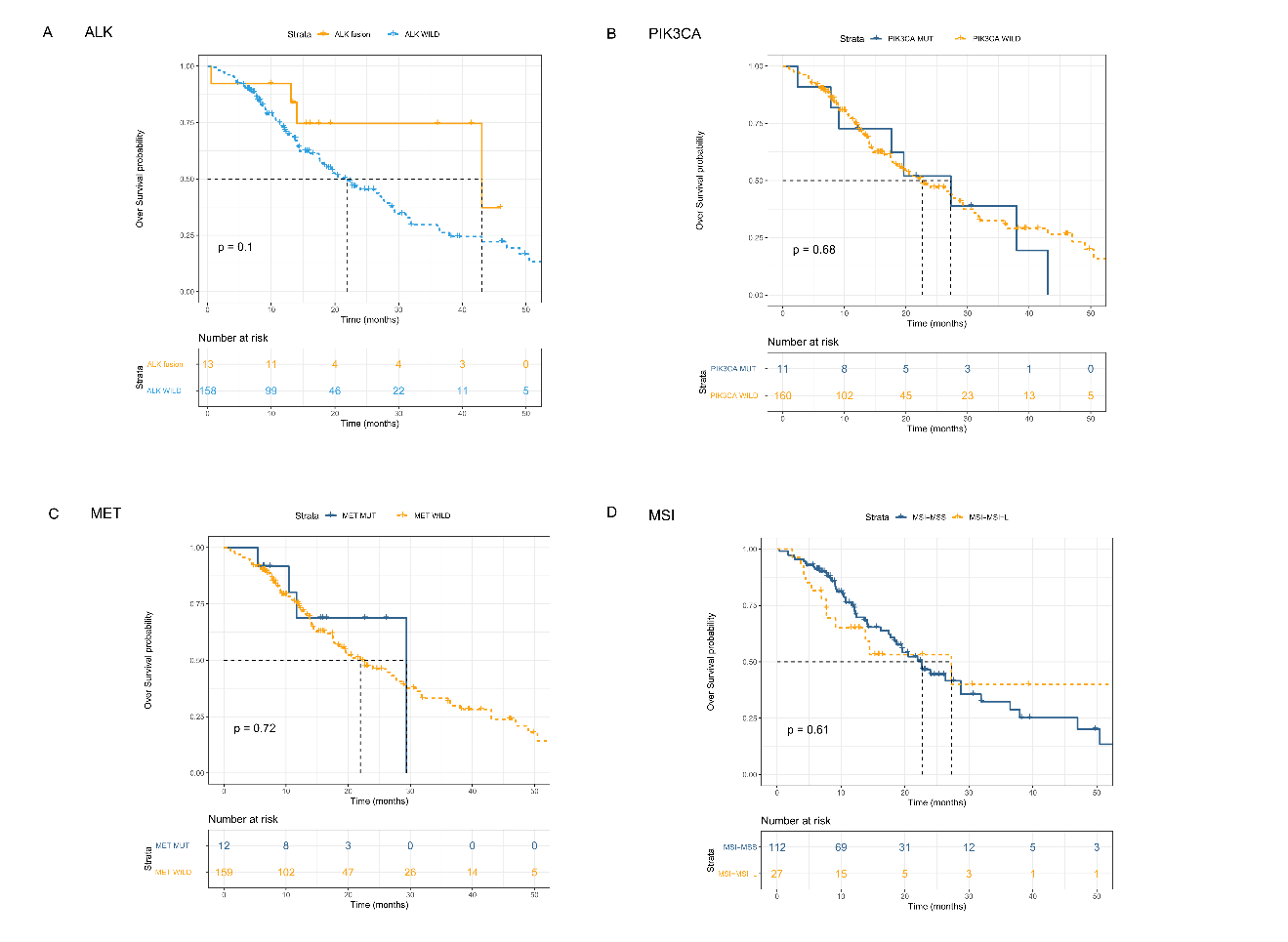


Figure S4. Overall survival of patients stratified by presence of mutation in specified gene
